# Regorafenib monotherapy or combined with an immune-checkpoint inhibitor as later-line treatment for metastatic colorectal cancer: a multicenter, real-world retrospective study in China

**DOI:** 10.1186/s12885-023-11700-w

**Published:** 2024-01-02

**Authors:** Wang Qu, Zimin Liu, Xiaobing Chen, Bo Liu, YunBo Zhao, Hao Yan, Xiujuan Qu, Shengmian Li, Aimin Zang, Yongkun Sun, Liangjun Zhu, Aiping Zhou

**Affiliations:** 1 Department of Medical Oncology, National Clinical Research Center for Cancer/Cancer Hospital, National Cancer Center, Chinese Academy of Medical Sciences and Peking Union Medical College, Beijing, 100021 China; 2 Department of Medical Oncology, Affiliated Hospital of Qingdao University, Qingdao, 266000 China; 3 Department of Medical Oncology, Affiliated Cancer Hospital of Zhengzhou University, Henan Cancer Hospital, Zhengzhou, 450003 China; 4 Department of Medical Oncology, Shandong Cancer Hospital, Jinan, 250117 China; 5 Department of Oncology, Beijing Hospital, National Center of Gerontology, Beijing, 100730 China; 6 Department of Oncology, Tianjin Union Medical Center, Tianjin, 300122 China; 7 Department of Oncology, The First Hospital of China Medical University, Shenyang, 110001 China; 8 Department of Gastroenterology and Hepatology, Fourth Hospital of Hebei Medical University, Shijiazhuang, 050011 China; 9 Department of Oncology, Affiliated Hospital of Hebei University, Baoding, 071000 China; 10 Department of Medical Oncology, Jiangsu Cancer Hospital, NO,42 Bai Zi Ting, Xuanwu District, Nanjing, 210000 China

**Keywords:** Colorectal cancer, Regorafenib, Immunotherapy

## Abstract

**Objective:**

To evaluate the efficacy and safety of regorafenib monotherapy or in combination with immune-checkpoint inhibitor while treating Chinese patients with metastatic colorectal cancer (mCRC): a real-world study.

**Methods:**

The data of patients with metastatic colorectal cancer who received regorafenib-containing regimen as the third or later line treatment at ten Chinese hospitals from Aug 2017 to Jun 2020 were retrospectively analyzed, including dosing details, survival data as well as adverse events. Survival analysis was further performed for patients administrated with regorafenib monotherapy and combined with an immune-checkpoint inhibitor based on Kaplan-Meier and Cox regression methods. The primary endpoint was overall survival.

**Results:**

A total of 537 patients were included with a median age of 61, among whom 376 received regorafenib monotherapy and 245 received regorafenib combined with immune-checkpoint inhibitors. The clinicopathological characteristics of the two groups at baseline were mainly balanced. No significant difference in progression-free survival (PFS) was observed in patients receiving regorafenib monotherapy or combination therapy (3.8 vs. 5.5 months, *p* = 0.170). In contrast, patients receiving combination therapy had a more prolonged overall survival (OS) than those receiving regorafenib monotherapy (13.5 vs. 10.0 months, *p* = 0.001). The treatment regimen and regorafenib dosage were significant prognostic factors in the multivariate analysis. Significant benefits in PFS and OS were achieved in *KRAS* mutant and anti-angiogenesis treatment-naïve subgroups receiving combination therapy compared to monotherapy. No apparent increase was recorded in treatment-related adverse events in patients receiving combination therapy.

**Conclusion:**

Regorafenib plus an immune-checkpoint inhibitor has already been a widely adopted strategy in the later-line treatment for mCRC in the real world. The combination therapy yielded a significantly prolonged overall survival than regorafenib alone, with a manageable safety profile in Chinese patients, and warrants further investigation.

**Trial registration:**

ClinicalTrials.gov Identifier: NCT04835324. Registered 6th April 2021.

## Background

Colorectal cancer is the third most frequently diagnosed cancer and the second most common cause of cancer-related death worldwide in 2020, with approximately 1.9 million new cases and 0.9 million deaths [1]. As one of the most common cancer, colorectal cancer also ranked second in incidence and fifth in mortality among all malignant tumors in China, according to the report from the National cancer center, with approximately 592 thousand new cases and 309 thousand deaths [2].

Regorafenib is a multitarget tyrosine kinase inhibitor against the vascular endothelial growth factor receptor (VEGFR) axis and alternative signal pathways. It thus suppresses tumor proliferation, metastasis, angiogenesis, and immune escape to exert anti-tumor efficiency [3]. Regorafenib is the current standard third-line treatment of colorectal cancer in China based on phase III trials as CORRECT and CONCUR. Combined with the results from several real-world studies, the overall survival for metastatic colorectal cancer patients receiving regorafenib monotherapy is 6.4 to 9.8 months, which remains unsatisfactory [4–7]. There is still an unmet need to further optimize the salvage-line therapies for mCRC.

Immune checkpoint inhibitor (ICI) achieved excellent efficacy in treating DNA deficient mismatch repair/microsatellite instability-high (dMMR/MSI-H) colorectal cancer and has become the standard first-line treatment nowadays based on the results from KEYNOTE 177 study [8]. While the MMR-proficient/microsatellite stable (pMMR/MSS) mCRC constitutes nearly 95% of mCRC, the response to immunotherapy remains unsatisfied. REGONIVO study reported by Shitara K showed an excellent overall response rate (ORR) of 33% and PFS of 7.9 months in 24 MSS mCRC patients receiving combination therapy of regorafenib and ICI [9]. Although such excellent efficacy hadn’t been reproduced in the subsequent phase II trials and retrospective studies adopting the same treatment modality with an ORR of 7 to 15% and mOS of 11.1 to 15.5 months, the results were still encouraging compared to the ORR of approximately 4% in patients receiving regorafenib monotherapy [10–12]. While phase III studies on a large scale are still warranted to validate further the survival benefits of combination therapy.

Due to the limited response of regorafenib monotherapy, combining regorafenib with ICIs is expected to be an optional strategy as a later-line treatment for mCRC while the OS benefit remains uncertain. Therefore, we designed this real-world study to analyze the treatment modality of regorafenib in the later-line treatment of mCRC, with an attempt to explore further whether the combination of regorafenib and ICIs could bring about additional survival benefits.

## Method

### Study design

This real-world study was conducted on mCRC patients who received regorafenib-containing treatment at ten hospitals, including the Cancer Hospital of the Chinese Academy of Medical Science, from Aug 2017 to Jun 2020 and was retrospectively reviewed. The study was approved by the ethics committee of the Cancer Hospital of the Chinese Academy of Medical Science, and the registration number at clinicaltrail.gov was NCT04835324.

The main inclusion criteria were: (1) cytologically or pathologically confirmed colorectal adenocarcinoma; (2) advanced unresectable metastatic disease; (3) failure with at least two standard treatments; (4) received at least one cycle of regorafenib-containing regimen;

The main exclusion criteria were: (1) participating in other interventional clinical trials during the regorafenib treatment; (2) the presence of other malignant tumors within five years before receiving regorafenib treatment, except for locally curable cancer (cured malignant melanoma, basal cell carcinoma, carcinoma in situ of bladder and cervix); (3) previous treatment of regorafenib in the first or second line.

### Outcome assessment

The primary endpoint was OS; other endpoints included progression-free survival and safety. OS was defined as the time from initiating regorafenib-containing treatment to death from any cause. PFS was defined as the time from starting regorafenib-containing therapy to disease progression or death for any reason, whichever occurred first. Adverse event (AE) was graded according to the National Cancer Institute Common Termiy Criteria for Adverse Events Version 4.03 (NCI-CTCAE 4.03).

### Statistical considerations

Statistical analysis was performed by SAS software version 4.1.1 and Stata software version 9. Continuous variables were compared by the Student’s t-test/Wilcoxon rank sum test or the Kruskal-Walis H test. Categorical data were compared using chi-square tests or Fisher’s exact test. Tumor measurements were compared with the implementation of nonparametric Mann–Whitney tests. The Kaplan-Meier method plots survival curves and calculates the median PFS and OS. Univariate and multivariate Cox Proportional Hazards Regression (CoxPH) model survival analyses were performed to identify prognostic factors. All the tests were two-tailed, and *p* values < 0.05 were considered to be statistically significant.

## Results

### Patient characteristics

**Fig. 1:**
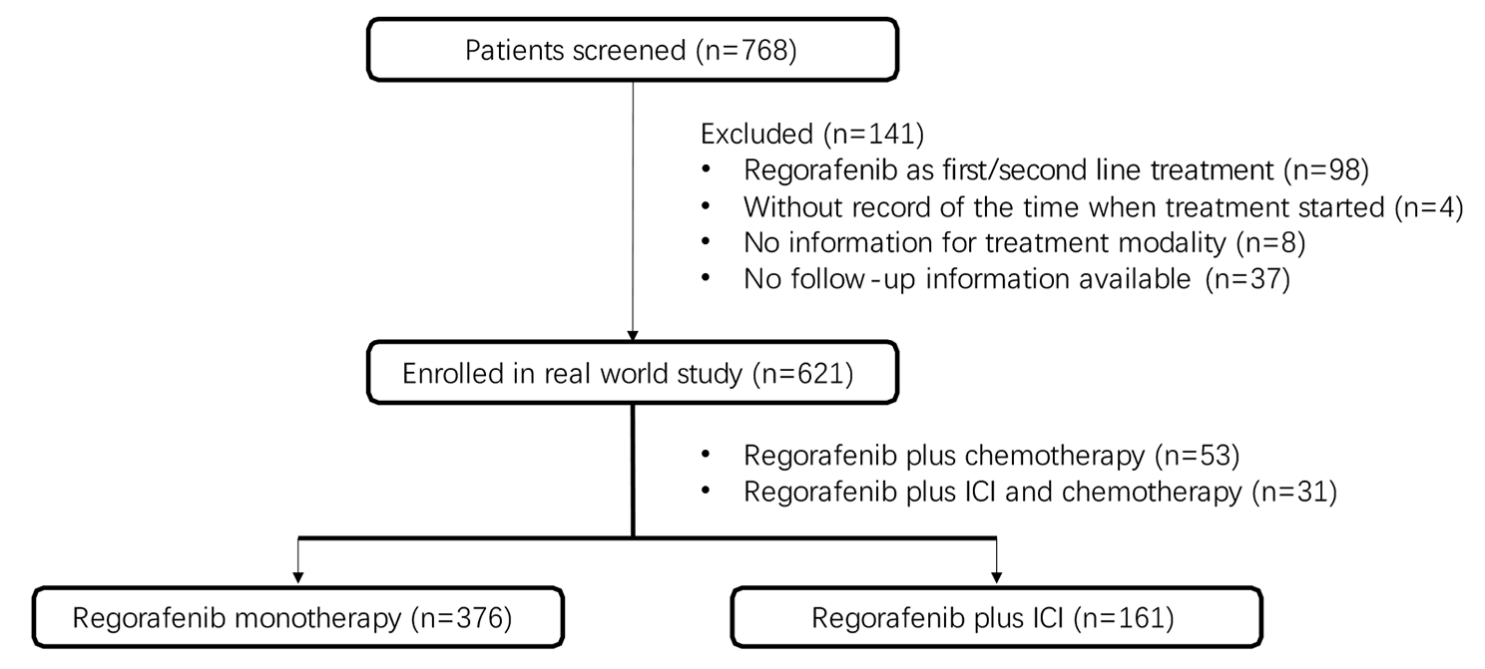
Flowchart depicting the patient selection process for the study

Seven hundred sixty-eight mCRC patients were screened; 98 received regorafenib as the first or second-line treatment and 49 patients without adequate clinical data were ruled out (Fig. 1). Five hundred thirty-seven patients were finally enrolled, with a median age of 61 at diagnosis (range:18 to 84). 366 (68.2%) patients were male; the most common metastatic site was the liver (48.4%). Regorafenib monotherapy was adopted in 376 (70.0%) patients, while the rest, 161 (30.0%), received regorafenib combined with ICI. Baseline characteristics, including age, gender, location of the primary lesion, MMR, and *RAS* gene status, were almost well balanced (Table 1). Among patients who received regorafenib plus ICI therapy, there was a higher proportion of cases with a history of previous target therapy than those who obtained regorafenib alone.

**Table 1:** Clinicopathological characteristics of patients receiving regorafenib monotherapy and regorafenib plus ICI

	Regorafenib(n = 376)	Regorafenib + ICI(n = 161)	*P* value
**Age**			0.143
Median (range)	61 (18–84)	59 (30–81)	
Mean ± SD	59.7 ± 11.3	58.1 ± 11.3	
**Gender**			0.118
Male	264 (70.2%)	102 (63.4%)	
Female	112 (29.8%)	59 (36.6%)	
**ECOG**			0.178
0/1	357 (95.0%)	157 (97.5%)	
2/3	19 (5.0%)	4 (2.5%)	
**Primary site**			0.734
Left-sided	257 (68.3%)	115 (71.4%)	
Right-sided	51 (13.6%)	25 (15.5%)	
Unknown	68 (18.1%)	21 (13.1%)	
**Liver metastasis**			0.859
Without	196 (52.1%)	81 (50.3%)	
With	180 (47.9%)	80 (49.7%)	
**MMR/MS**			0.082
Available	192 (51.1%)	79 (49.1%)	
dMMR/MSI-H	10 (5.2%)	0	
pMMR/MSS	182 (94.8%)	79 (100.0%)	
Unavailable	184 (48.9%)	82 (50.9%)	
***RAS *** **gene status**			0.251
Available	236 (62.8%)	118 (73.3%)	
Wild type	113 (47.9%)	49 (41.5%)	
*KRAS* mutated	116 (49.2%)	62 (52.5%)	
*NRAS* mutated	7 (3.0%)	7 (5.9%)	
Unavailable	140 (37.2%)	43 (26.7%)	
***BRAF *** **gene status**			0.082
Available	170 (45.2%)	73 (45.3%)	
Wild type	163 (95.9%)	65 (89.0%)	
V600E mutated	7 (4.1%)	8 (11.0%)	
Unavailable	206 (54.8%)	88 (54.7%)	
**Prior treatment**			
Median lines	3 (3–8)	3 (3–7)	
Anti-VEGFR	233 (62.6%)	103 (64.0%)	0.768
Anti-EGFR	32 (8.6%)	15 (9.3%)	0.789
Anti-VEGFR & EGFR	51 (13.7%)	32 (19.9%)	0.071
No targeted therapy	56 (15.1%)	11 (6.8%)	0.009
Unknown	4 (1.1%%)	0	

The daily maintenance dose of regorafenib was collected in 340 (63.3%) patients. In the group of regorafenib monotherapy, 75 (34.4%) patients received 80 mg as the daily maintenance dose, 81 (37.2%) patients received 120 mg, and 61 (28.0%) patients received 160 mg. While in the group of regorafenib plus ICI, 68 (55.7%) patients received 80 mg, 30 (24.6%) patients received 120 mg, and 23 (18.9%) patients received 160 mg. Moreover, 145 (90.1%) of 161 patients receiving combination therapy had the specific medication of ICI recorded. A total of 5 programmed death-1 (pd-1) inhibitors were adopted. 45 (31.0%) patients received Sintilimab treatment, 37 (25.5%) received Toripalimab, 35 (24.1%) received Camrelizumab, 7 (4.8%) received Tieslelizumab, and 6 (4.1%) received Pembrolizumab.

### Survival analysis

**Fig. 2:**
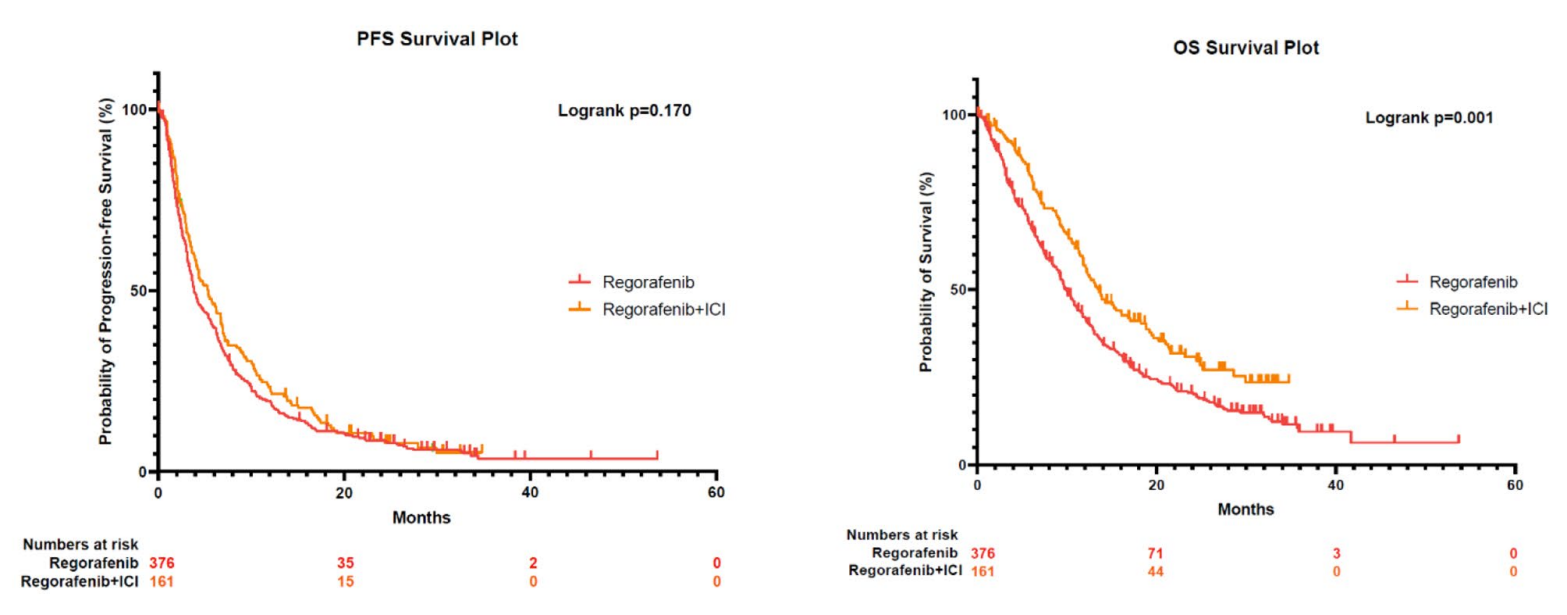
PFS and OS plots for patients receiving regorafenib monotherapy and regorafenib plus ICI

As of March 31, 2022, the median follow-up time was 28.4 months (95%CI: 25.9–30.8 months). The median treatment time was 3.2 months and 4.0 months for patients receiving regorafenib and regorafenib plus ICI. The median PFS was 3.8 and 5.4 months, respectively (*p* = 0.170), with no statistical difference. While patients receiving regorafenib plus ICI had remarkably prolonged median OS compared to those receiving regorafenib alone (13.5 vs. 10.0 months, *p* = 0.001) (Fig. 2). Univariate analysis revealed that prior treatment lines, mono or combination therapy, and treatment centers were prognostic factors. While in multivariate analysis, only treatment modality and maintenance dosage could affect survival significantly (*p* = 0.017 and 0.005, respectively) (Table 2). Patients receiving regorafenib plus ICI and those with a higher maintenance dosage of regorafenib, like 160 mg, had more prolonged survival. Besides, patients with lower Eastern Cooperative Oncology Group (ECOG) scores also tended to have more prolonged survival (11.2 vs. 9.7 months, *p* = 0.056).

**Table 2:** Uni- and multivariate survival analysis for patients receiving regorafenib monotherapy and regorafenib plus ICI

Variable	OS, months (95% CI)	Univariate P value	HR	95% CI	Multivariate *P* value
Age	11.17 (10.08–12.26)	0.542	1.01	0.99–1.03	0.238
Gender		0.288	1.29	0.88–1.90	0.195
Female	11.36 (9.17–13.57)				
Male	10.84 (9.50-12.18)				
ECOG		0.141	2.26	0.98–5.21	0.056
0/1	11.20 (10.13–12.28)				
2/3	9.66 (1.53–17.78)				
*RAS*		0.254	0.95	0.68–1.33	0.780
*RAS* WT	13.57 (11.02–16.12)				
*KRAS* MT	10.91 (9.43–12.39)				
*NRAS* MT	9.56 (3.12-16.00)				
Primary Location		0.955	1.44	0.94–2.20	0.093
Left-side	11.73 (10.47–12.99)				
Right-side	9.20 (5.21–12.48)				
Liver Metastasis		0.088	0.73	0.47–1.12	0.146
With	10.84 (9.22–12.47)				
Without	11.50 (9.26–13.73)				
Pulmonary Metastasis		0.321	1.07	0.57–2.03	0.833
Pulmonary-limited	13.01 (10.41–15.61)				
Extrapulmonary	10.81 (9.40-12.22)				
Prior Treatment		0.781	0.90	0.73–1.10	0.300
Anti-VEGFR	4.14 (3.37–4.91)				
Anti-EGFR	3.29 (2.89–3.68)				
Anti-VEGFR & EGFR	5.45 (3.81–7.10)				
Naïve	4.67 (1.77–7.56)				
Pattern		0.001	0.77	0.62–0.95	0.017
R	9.99 (8.90-11.07)				
R + I	13.47 (11.21–15.73)				
Maintenance dose		0.081	0.71	0.55–0.90	0.005
40 mg	2.92				
80 mg	11.07 (8.47–13.67)				
120 mg	12.03 (9.81–14.24)				
160 mg	15.67 (10.83–20.51)				

**Fig. 3:**
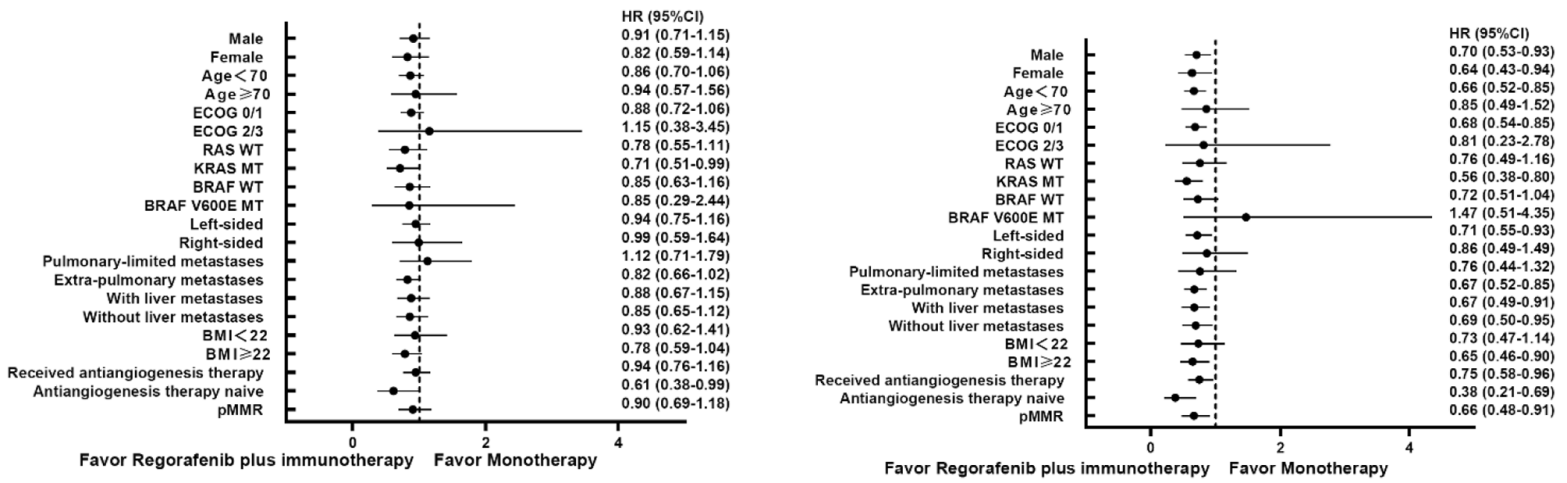
Subgroup analysis for patients receiving regorafenib monotherapy and regorafenib plus ICI

Subgroup analysis presented no significant difference in PFS in terms of different gender, ages, ECOG scores, location of the primary lesion, and metastatic status between patients receiving monotherapy or combination therapy (Fig. 3). In contrast, patients with *KRAS* mutation or without prior anti-angiogenesis treatment achieved a longer PFS when receiving combination therapy compared to regorafenib alone (6.8 vs. 3.7 months, HR 0.71 [0.51–0.99]; 6.7 vs. 3.3 months, HR 0.61 [0.38–0.99]). Longer OS were observed in most subgroups favoring combination therapy, including patients <70 years old, ECOG 0–1, Body Mass Index (BMI) ≥ 22, liver metastasis, and pulmonary metastasis. A more prolonged OS was also reached in patients with *KRAS* mutation or without prior anti-angiogenesis therapy receiving combination therapy (13.0 vs. 9.2 months, HR 0.56 [0.38–0.80]; 21.3 vs. 10.1 months, HR 0.38 [0.21–0.69]).

In patients treated with regorafenib combined with ICI, the PFS were 6.1, 10.6, and 11.2 months for patients with a daily maintenance dose of 80 mg, 120 mg, and 160 mg (*p* = 0.051) respectively, and the OS was 12.6, 13.9 and 23.1 month (*p* = 0.130) respectively. Although without a statistical difference, a trend of prolonged PFS and OS favoring higher maintenance daily dose was observed.

### Safety

In the safety analysis, 58 (10.8%) were excluded due to the loss of record for adverse event. In the rest 479 patients, adverse events of any grade occurred in 69.1% of patients. The most common AE was hand-foot syndrome, with an incidence of 20.5%. Other AEs occurring in 15% or more of patients were elevated bilirubin (17.7%), anemia (16.9%), fatigue (17.3%), and elevated aspartate aminotransferase (16.7%). Moreover, 2 (0.4%) patients developed hypothyroidism, and 2 (0.4%) patients developed interstitial pneumonia. No treatment-related death occurred. For patients administrated with regorafenib monotherapy, adverse events of any grade occurred in 66.3% (222/355), while grade 3 or 4 treatment-related adverse events occurred in 15.2% (51/355) of patients. For patients administrated with regorafenib plus ICI, the occurrence of adverse events of any grade was 87.8% (109/124) and 13.7% (12/124) for grade 3 or 4 treatment-related adverse events. Specific treatment-related adverse events by cohort are shown in Table 3.

**Table 3:** Treatment-related adverse events in patients receiving regorafenib monotherapy and regorafenib plus ICI

AE, all grades	Regorafenib (n = 355)	Regorafenib + ICI (n = 124)	AE, all grades	Regorafenib (n = 355)	Regorafenib + ICI (n = 124)
Hand-foot syndrome	63 (17.7%)	35 (26.6%)	Anorexia	34 (9.6%)	16 (12.9%)
Total bilirubin increased	60 (16.9%)	25 (20.2%)	Hypertension	17 (4.8%)	17 (13.7%)
Anemia	50 (14.1%)	31 (25.0%)	Neutropenia	15 (4.2%)	15 (12.1%)
Aspartate aminotransferase increased	55 (15.5%)	25 (20.2%)	Nausea	17 (4.8%)	12 (9.7%)
Fatigue	55 (15.5%)	28 (22.6%)	Abdominal pain	12 (3.4%)	11 (8.9%)
Alanine aminotransferase increased	37 (10.4%)	23 (18.5%)	Rash	8 (2.3%)	11 (8.9%)
Thrombocytopenia	33 (9.3%)	26 (21.0%)	Fever	10 (2.8%)	9 (7.3%)
Leukopenia	23 (6.5%)	23 (18.5%)	Arthralgia	9 (2.5%)	8 (6.5%)

Note: Data are presented as n (%)

## Discussion

As a real-world study, regorafenib monotherapy brought about a PFS of 3.8 months and an OS of 10.0 months, comparable to the results of prior phase III clinical trials and other large-scale real-world studies [4, 5]. Since the improvements of OS were only 1.4 to 2.5 months for regorafenib monotherapy compared to best supportive care in randomized studies, which was relatively limited, the optimization of later line treatment in mCRC was urgent, and the combination of regorafenib and ICI was one of the potential strategies. Our study had no statistical difference in PFS between combination therapy and monotherapy (5.5 vs. 3.8 months, *p* = 0.170), while the OS for combination therapy achieved 13.5 months, significantly superior to monotherapy (*p* = 0.001). The OS of combination therapy in this study was consistent with those of 10.8 to 15.5 months in other studies with small samples, including REGOMUNE [11, 12]. However, most prior studies were single-arm designed, and no direct comparison with regorafenib monotherapy was performed. The multivariate analysis in our study demonstrated that combination therapy was an independent favorable prognostic factor for OS. Thus, the results indicated that the combination of regorafenib and ICI was a promising strategy in the later-line treatment of mCRC and warranted further investigation in the Chinese population. The phase III study LEAP-017 comparing lenvatinib plus pembrolizumab with regorafenib is expected to provide a precise answer [13].

The mechanism for the advantages of combination therapy over monotherapy may result from the synergistic interaction between anti-angiogenesis and immunotherapy. Several preclinical studies of regorafenib combined with ICI demonstrated that regorafenib could reduce the infiltration of immunosuppressive macrophages and regulatory T (Treg) cells in the tumor microenvironment. At the same time, ICI could elevate the intratumor level of interferon γ. The synergistic interaction could induce the M1 polarization of macrophages, resulting in dual inhibition of Treg cells and a prominent anti-tumor effect [14–16]. While the limited improvement in PFS might attribute to the feature of immunotherapy, which could prolong survival even without obvious tumor regression. At the same time, the capability of immunotherapy to induce long-term tumor regression may also remarkably elongate of overall median survival. Similar results were reported in the CO.26 study [17].

Subgroup analysis in our study further revealed that combination therapy brought more survival benefits in patients with younger age, better physical status, *KRAS* mutant or anti-angiogenesis treatment naïve. In studies with small sample sizes, patients with *KRAS* mutation receiving regorafenib monotherapy presented relatively poor efficacy. In addition, subgroup analysis in the CORRECT trial also showed no significant survival benefits in patients with *KRAS* mutation receiving regorafenib compared to those receiving the best supportive care. While in our study, the mPFS and mOS were 6.8 and 13.0 months in *KRAS* mutant patients administrated with regorafenib plus ICI, significantly longer than those receiving monotherapy with mPFS of 3.7 and mOS of 9.3 months (*p* = 0.041 and *p* = 0.002, respectively). This result implied that combination therapy might bring more benefits in the later-line treatment for MSS mCRC with *KRAS* mutation compared to monotherapy. Similar superiority was also reported in 96 *KRAS* mutant patients enrolled in another retrospective study, as the treatment of regorafenib combined with anti-PD-1 antibodies showed a longer mPFS than regorafenib monotherapy (HR = 0.59, 95%CI: 0.37–0.96) [18].

Several previous studies consistently indicated that patients with liver metastasis responded poorly to regorafenib and ICI combination therapy, with an ORR of 0 to 8.7%. Besides, similar PFS was achieved in patients with liver metastasis receiving regorafenib monotherapy or combination therapy (2.0 vs. 2.0 months, *p* = 0,779) in a retrospective study. Interestingly, although no difference in PFS was observed between patients with liver metastasis receiving monotherapy and combination therapy in our study, overall survival benefits were achieved in favor of combination therapy. Even though the liver bears an immunosuppressive microenvironment, the combination of anti-angiogenesis and immunotherapy seemed to slow down the progression of the tumor, probably by increasing local lymphocyte infiltration and improving the overall immunity function of the body, which finally resulted in the prolonged patient’s survival. Further validation through a prospective study was required to verify this result.

The maintenance dose was a prognostic factor in the multivariate analysis in our study, and higher dosage was correlated with longer OS. The overall survivals of patients receiving regorafenib with 120 mg and 160 mg daily were numerically longer than patients receiving 80 mg daily in the combination treatment group. However, no statistical difference was observed (13.9 and 23.1 vs. 12.6 months). In another prospective phase Ib study of regorafenib plus toripalimab for mCRC, a high incidence of severe hand-foot syndrome occurred at the dose of 120 mg, and the final recommended dose of regorafenib for combination therapy was 80 mg, which was also the most commonly adopted dose in 55.7% of the patients in our study [10].

For the safety profiles, the incidence of the overall adverse events was lower than the data in previous studies of regorafenib monotherapy. At the same time, common toxicities, including hand-foot syndrome and fatigue, were consistent with the historical data, up to approximately 30%. The immune-related toxicities were lower than those reported in several large-scale clinical trials, such as thyroid dysfunction (0.4% vs. 5.0%) and interstitial pneumonia (0.4% vs. 3.6%). The incidence of all grade adverse events was higher in the combination treatment compared to monotherapy (87.9% vs. 66.3%, p<0.05), while the incidences of grade 3/4 toxicities were similar in two groups (15.2% vs. 13.7%, *p* = 0.685). As a real-world study, a considerable bias probably exists due to the incomplete record of adverse events, which is also a shortcoming of this study.

This study still presents several limitations. First, as a retrospective actual world study, the bias of patient selection between two groups and the heterogenous quality control among individual study centers could hardly be evitable. Secondly, insufficient data collection led to the failure of further analysis for the dosage adjustment of regorafenib as well as subsequent treatment. Thirdly, discrepancy and loss in radiological data also resulted in insufficiency in tumor response evaluation. Last but not least, the incomplete data on safety impeded the further investigation between adverse events and efficacy in our study.

## Conclusions

In conclusion, the combination of regorafenib and ICI has already been widely adopted in the later-line treatment for mCRC in the real world. Regorafenib combined with an ICI yielded a significantly prolonged overall survival than regorafenib alone, with a manageable safety profile in Chinese patients. 80 mg daily could be the most acceptable dose of regorafenib when combined with an ICI. The combination strategy warrants further verification in a prospective phase III trial.

## Data Availability

The datasets used and/or analysed during the current study are available from the corresponding author on reasonable request.

## Abbreviations

mCRC: Metastatic colorectal cancer
PFS: Progression-free survival
OS: Overall survival
VEGFR: Vascular endothelial growth factor receptor
ICI: Immune checkpoint inhibitor
dMMR/MSI-H: Deficient /microsatellite instability-high
pMMR/MSS: Mismatch repair-proficient/microsatellite stable
ORR: Overall response rate
AE: Adverse event
PD-1: Programmed death-1
ECOG: Eastern Cooperative Oncology Group
BMI: Body Mass Index
